# The dopamine β-hydroxylase gene in Chinese goose (*Anas cygnoides*): cloning, characterization, and expression during the reproductive cycle

**DOI:** 10.1186/s12863-016-0355-8

**Published:** 2016-02-24

**Authors:** Qi Xu, Yadong Song, Ran Liu, Yang Chen, Yang Zhang, Yang Li, Wenming Zhao, Guobin Chang, Guohong Chen

**Affiliations:** Key Laboratory of Animal Genetics and Breeding and Molecular Design of Jiangsu Province, Yangzhou University, Yangzhou, 225009 PR China; School of Animal science and Technology, Yangzhou University, Yangzhou, PR China

**Keywords:** Goose, *DBH*, Gene expression, Reproduction

## Abstract

**Background:**

Dopamine β-hydroxylase (DBH) is a critical enzyme in the biosynthesis of catecholamines. This enzyme’s role in neuroendocrine regulation is well known, but there are some indications that it may also modulate reproduction and endocrine in mammals and birds. We selected goose (*Anas cygnoides*) as an ideal model species for investigating the role of *DBH* in avian reproduction.

**Results:**

Full-length cDNA encoding DBH was cloned from Zhedong goose using reverse transcription PCR and rapid amplification of cDNA ends. The cDNA consisted of a 126-base pair (bp) 5′-untranslated region (UTR), a 379-bp 3′-UTR, and an 1896-bp open reading frame encoding a polypeptide of 631 amino acids. The deduced amino acid sequence of gDBH shared high homology with an analogue from other birds and contained three conserved domains from a mono-oxygenase family including a DOMON domain and two Cu2_mono-oxygen domains. Real-time quantitative PCR analysis showed that *gDBH* mRNA was expressed in both reproductive and endocrine tissues of Zhedong goose, specifically in the hypothalamus, pituitary, ovary, and oviduct. More DBH mRNA of reproductive and endocrine tissues was detected at ovulation than at oviposition in Zhedong goose. Evidence of opposite trend of *gDBH* expression was found between the hypothalamus-pituitary and oviduct during the ovulation phase and the broody phase. In addition, we assessed DBH mRNA expression during ovulation in two breeds of geese that differ in egg production. The reproductive and endocrine tissues of Yangzhou geese with higher egg production had more gDBH expression than Zhedong geese. Finally, the five non-synonymous SNP(c.1739 C > T, c.1760G > T, c.1765A > G, c.1792 T > C and c.1861G > C) were identified in the coding region of DBH gene between Zhedong goose and Yangzhou goose.

**Conclusions:**

We conclude that goose *DBH* mRNA show obvious periodically variation in reproductive and endocrine tissues during the reproductive cycle in geese.

**Electronic supplementary material:**

The online version of this article (doi:10.1186/s12863-016-0355-8) contains supplementary material, which is available to authorized users.

## Background

Dopamine β-hydroxylase (DBH) catalyzes the conversion of dopamine to norepinephrine in the biosynthesis of catecholamines [1–3]. The activity of DBH influences the levels of dopamine and the biosynthesis of norepinephrine and epinephrine. Dopamine β-hydroxylase’s importance in the nervous system is well established [4–9], but a few studies also point to its importance in reproduction. The evidence for this in mammals can be summarized as follows. DBH, regulated by the sympathetic nervous system, has major effects in the female reproductive system of pigs, where it influenced ovarian and oviductal function [10]. Injection of 1-phenyl-3-(2-thiazolyl)-2-thiourea (U-14,624), a DBH inhibitor, increased the number of progestin and estrogen receptors in female rat [11–13]. In research with other mammals, DBH regulated reproductive performance through modulating the concentration of catecholamines, as well as other physiological functions. Mice with targeted disruption of *DBH* had a high fetal mortality rate and altered maternal behavior [14, 15]. In pigs, polymorphism of *DBH* was related to reproduction and piglet survivability [16]. This background was intriguing and led us to speculate on the role of DBH in avian reproduction, about which little is known.

The goose (*Anas cygnoides*) is a commercially important food source that is widely cultivated in China. It is an ideal avian model for characterization of reproduction because of its obvious reproductive stages and strong broodiness [17]. In a previous study in which we used transcriptome profiling of ovaries from laying and brooding geese [18], we identified *DBH* as an important gene in the goose reproductive cycle. We have extended this study here by cloning the Zhedong goose *DBH* and characterized its spatio-temporal expression patterns by qPCR. Next, we undertook a correlative study of *DBH* expression and egg production by comparing *DBH* expression in the Yangzhou breed, which has high-egg production, with the Zhedong goose, a breed with low-egg production and strong broodiness behavior. The *DBH* expression profiles provide an invaluable information for understanding of the regulatory function of *DBH* in goose egg laying.

## Results

### Zhedong Goose DBH cDNA cloning and sequence analyses

The full-length cDNA of *gDBH* was acquired with RT-PCR and RACE. The *DBH* cDNA from Zhedong goose was 2399 nucleotides in length and consisted of a 126-nucleotide 5′ untranslated region (UTR), a 379-nucleotide 3′ UTR, and an 1896-nucleotide open reading frame (ORF) putatively encoding a single 631 amino acid protein(GenBank accession KU672379). The other transcript variant was not detected in Zhedong goose in this study.

### Phylogenetic analysis of the putative DBH

**Fig. 1:**
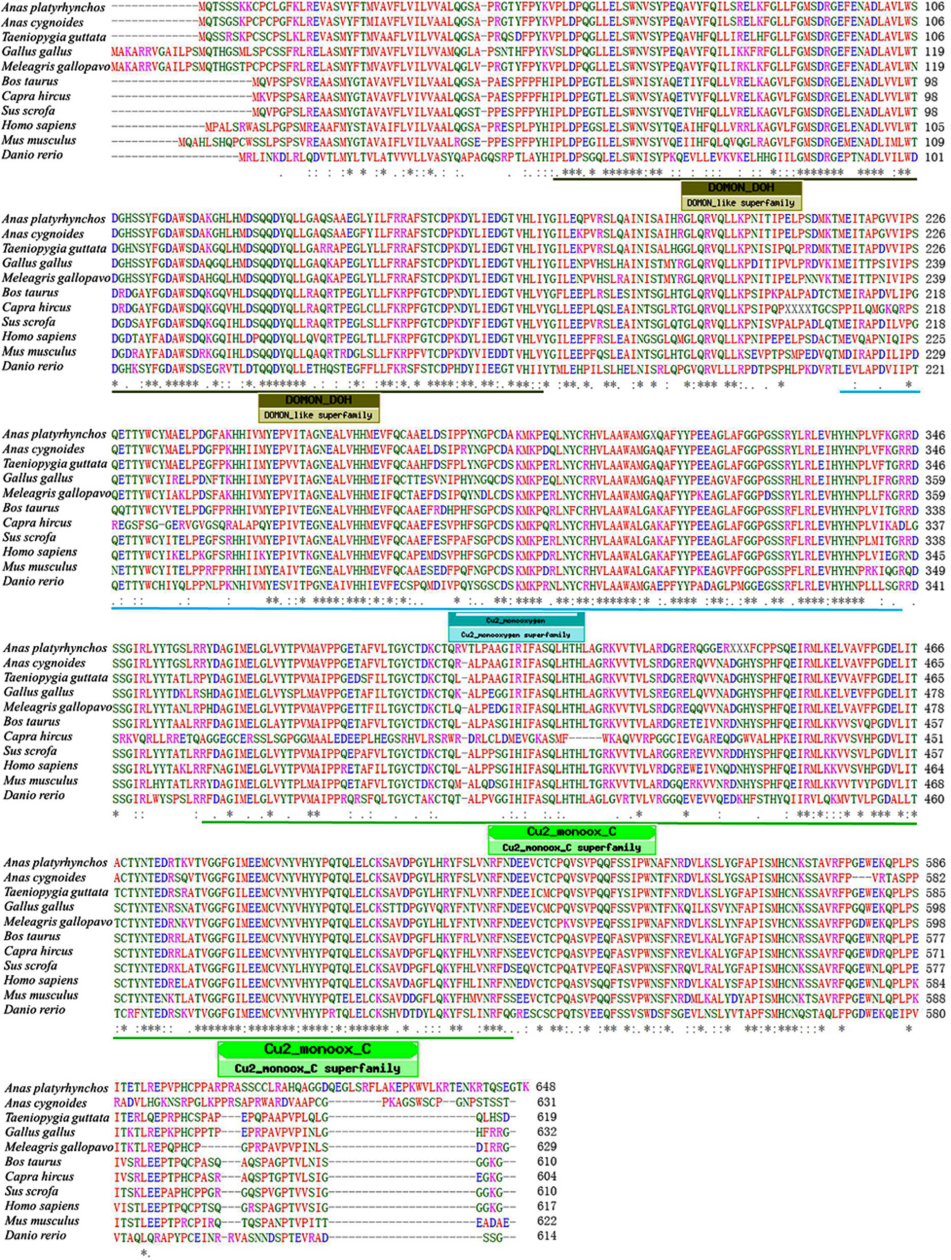
Multiple sequence alignment of the deduced amino acid sequence of gDBH with those of other species. All DBH protein sequences from 11 species were aligned by the Clustal W program. Additional GenBank accession numbers not mentioned elsewhere are as follows: chicken (*Gallus gallus*, XM_415429), duck (*Anas platyrhynchos*, XM_005013310), turkey (*Meleagris gallopavo*, XM_003211322), zebra finch (*Taeniopygia guttata,* XM_004174352), human (*Homo sapiens*, NM_000787), mouse (*Mus musculus*, NM_138942), pig (*Sus scrofa*, XM_001927211), cattle (*Bos taurus*, NM_180995), goat (*Capra hircus*, XM_005687352), zebra fish (*Danio rerio*, NM_001109694). Asterisk indicates residues that are identical among all species; dashes indicate gaps introduced to facilitate alignment; the underline indicates the conserved domains

Alignment analysis of the DBH protein (Fig. 1) revealed that the putative goose *DBH* had high homology with analogues from the other four birds (chicken, duck, turkey and zebra finch). There was less homology with the non-bird species than the avian species.

The structural domain of the DBH protein of different species was compared with gDBH. It is relatively conservative and contains three potential domains (a DOMON domain, goose DBH 52-170AA; the two Cu2_mono-oxygen domains in the N-terminal and C-terminal, respectively, goose DBH 215-344AA, 360-524AA), which belonged to a mono-oxygenase family. The conserved domains in gDBH and the amino acid sequence similarity with other DBHs strongly suggested that it was a homologue of DBH from *Anas cygnoides*.

**Fig. 2:**
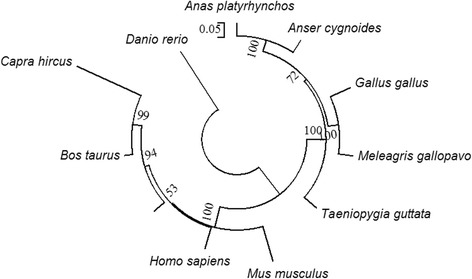
Phylogenetic tree based on the amino acid sequence of gDBH and other homologous sequences. The tree was constructed with Clustal W. The reliability of the neighbor-joining tree was estimated by bootstrap analysis with 1,000 replicates. Bootstrap values are shown on the lineages of the tree and major taxonomic clusters are indicated separately. The position of the root of the phylogenetic tree was established by using *Danio rerio* as an outgroup. The scale bar indicates 5 % amino acid divergence within a sequence

To determine the evolutionary relationship between gDBH and the other proteins, phylogenetic analysis was carried out by Clustal W and Mega 6.0. The amino acid sequences of gDBH and from another ten species were compared. Protein sequences were used for the rooted phylogenetic tree, which was constructed by the neighbor-joining method. The goose proteins from different species were divided into three major branches. *Gallus gallus*, *Anas platyrhynchos*, *Anas cygnoides*, *Meleagris gallopavo*, *Taeniopygia guttata* were grouped into a cluster. The second branch consisted of *Homo sapiens*, *Mus musculus*, *Sus scrofa*, *Bos taurus*, and *Capra hircus. Danio rerio* was separated and formed an independent branch (Fig. 2). The established evolutionary relationship tree was consistent with the real evolution of animals.

### Expression pattern of DBH in different tissues and reproductive cycle stages of Zhedong goose

**Fig. 3:**
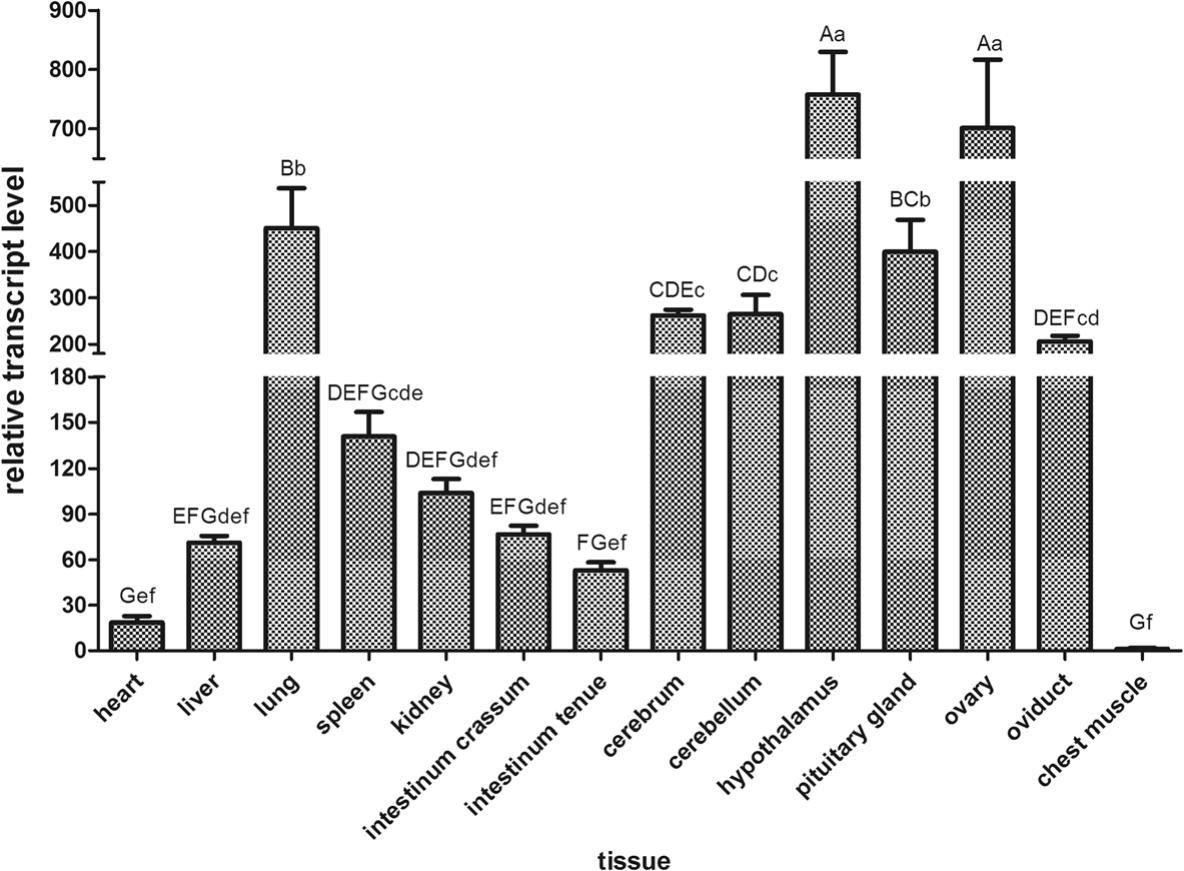
Expression of *gDBH* in various tissues of Zhedong geese. Gene expression was determined by qPCR and is represented relative to *GAPDH* expression. Vertical bars represent the mean ± S.D. (*n* = 3). Different capital letters above error bars indicate highly significant differences between means (*P* < 0.01). Different lower case letters indicate significant differences (*P* < 0.05)

The qPCR demonstrated that *DBH* was differently expressed in fourteen tissues of Zhedong goose. High levels of *DBH* transcript were detected in hypothalamus, pituitary, ovary, oviduct, lung, cerebrum, and cerebellum tissues in Zhedong goose while levels were negligible in chest muscle tissue (Fig. 3).

**Fig. 4:**
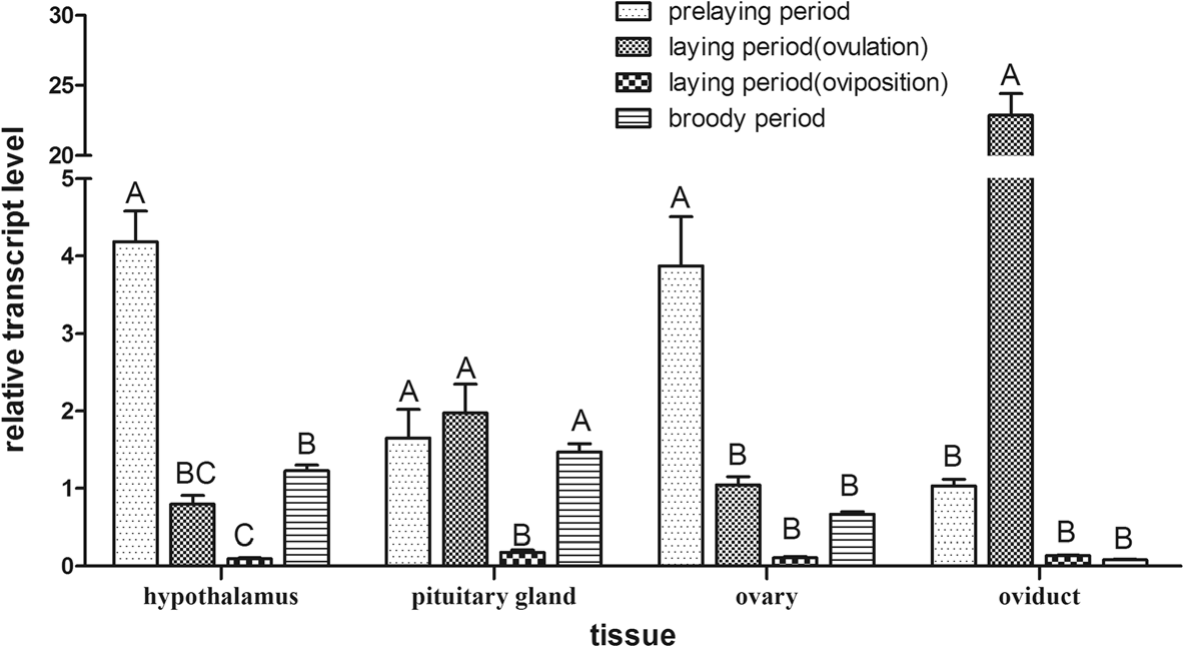
Expression patterns of *gDBH* during the reproductive cycle of Zhedong geese. Gene expression was determined by qPCR and is represented relative to *GAPDH* expression. Vertical bars represent the mean ± S.D. (*n* = 3). Different letters above error bars indicate highly significant differences between respective means (*P* < 0.01)

We wished to examine the temporal expression patterns of *gDBH* expression in the hypothalamus, pituitary, ovary, and oviduct tissues during different stages of the goose reproductive cycle, specifically the pre-laying stage, ovulation, oviposition, and the broody phase. The results are shown in Fig. 4. Overall, there were differences in *gDBH* expression with respect to both time and tissue. Expression was lowest in all tissues during the oviposition phase. It was high in the hypothalamus, ovary, and pituitary in the pre-laying period. Expression was higher in hypothalamus-pituitary than in the oviduct during the broody phase. In contrast, the expression of the *DBH* gene was high in the oviduct during ovulation phase, but low in the hypothalamus-pituitary.

### Comparison of DBH expression of Zhedong goose and Yangzhou goose during the ovulation phase

**Fig. 5:**
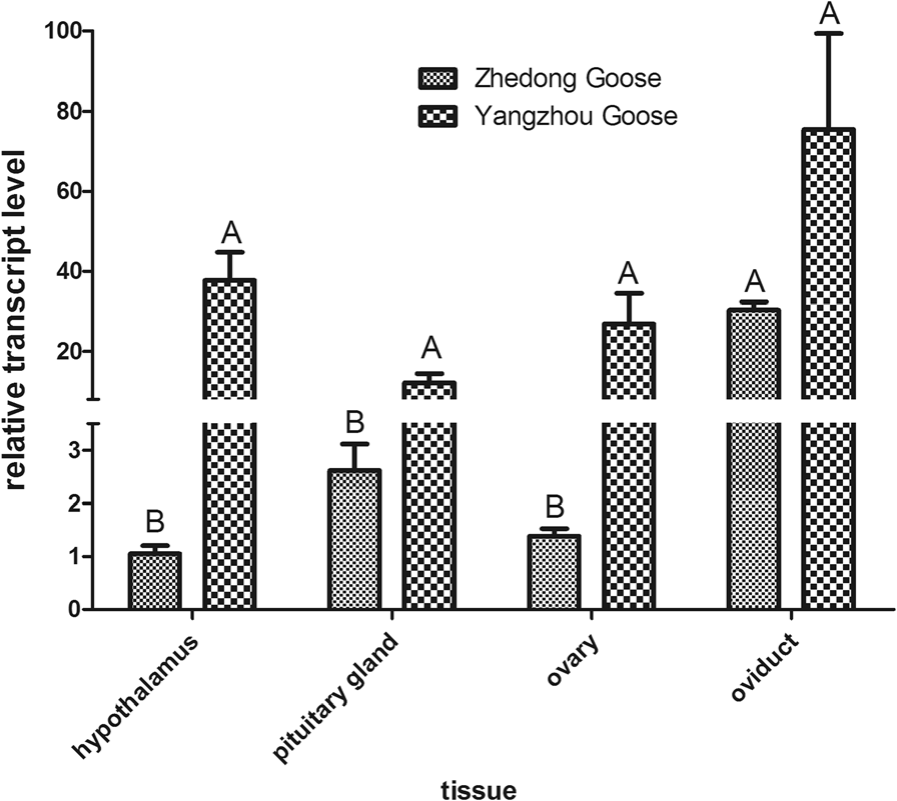
*DBH* expression in tissues of Zhedong geese and Yangzhou geese during ovulation. Gene expression was determined using qPCR and is represented relative to expression of *GAPDH*. Vertical bars represent the mean ± S.D. (*n* = 3). Different letters above error bars indicate highly significant differences between respective means (*P* < 0.01)

Two goose breeds, with markedly different egg performance, were selected to test the hypothesis that *DBH* expression might be correlated with egg production. The comparison was made between the Zhedong goose, with low egg production, and the Yangzhou goose, with high egg production. The expression of *DBH* was higher in reproductive and endocrine tissues of the high-egg-producing, Yangzhou goose than in the Zhedong goose during the ovulation phase. These differences were significant for the hypothalamus, pituitary, and ovary (*P* < 0.01), but not for the oviduct (Fig. 5).

### Identification of genetic variation on DBH in Zhedong goose and Yangzhou goose

By sequence alignment, the five nsSNPs (non-synonymous SNP) including c.1739 C > T, c.1760G > T, c.1765A > G, c.1792 T > C and c.1861G > C were identified in the Exon 11 of goose DBH gene (Table 1). The allele frequency from c.1739 C > T and c.1792 T > C substitutions were significantly different between Zhedong goose and Yangzhou goose (*P* < 0.01).

**Table 1:** Absolute frequencies of the five nsSNPs on DBH gene in Yangzhou goose and Zhedong goose

Breeds	c1739C > T				c1760G > T				c1765A > G				c1792T > C				c1861G > C			
	CC	CT	TT	*P* value	GG	GT	TT	*P* value	AA	AG	GG	*P* value	TT	TC	CC	*P* value	GG	GC	CC	*P* value
Yangzhou goose(*n* = 29)	12	12	5	0.008	4	9	16	0.02	0	9	20	0.03	2	6	21	0.01	0	4	25	0.07
Zhedong goose(*n* = 22)	4	5	13		1	1	20		5	5	12		9	3	10		0	0	22	

## Discussion

In this study, the goose *DBH* gene was characterized from *Anas cygnoides*. A DOMON domain and a Cu2_mono-oxygen domain were identified in the deduced amino acid sequence of gDBH. Both DOMON and Cu2_mono-oxygen domains were highly conserved in all the DBHs analyzed. The DOMON domain had been identified in the physiologically important enzymes including cellobiose dehydrogenase, extracellular fungal oxidoreductase and ethylbenzene dehydrogenase [19, 20]. Recent studies indicate that DOMON domains are responsible for heme or sugar recognition and binding at the cell surface [21] and it has been suggested to be a dopamine-binding domain in DBHs [22]. The Cu2_mono-oxygen domain may be the catalysis center of DBH and, as such, involved in the conversion of dopamine to norepinephrine. The conserved DOMON domain and Cu2_mono-oxygen domain identified in goose DBH in this study led us to speculate that it had the same function as other avian DBHs, namely, in the synthesis of catecholamines.

Quantitative PCR analysis revealed that *DBH* was expressed in every tissue analyzed except chest muscle, albeit to different degrees in different tissues. The information on the tissue distribution might provide clues about the role of DBH in various physiological functions in birds. The ubiquitous distribution of DBH that we observed in goose tissues had not been observed in mammalian tissues, where it was mainly present in the nervous system [5–7]. Of course, high expression of *DBH* was found in the cerebrum and cerebellum in goose, no doubt due to the involvement of norepinephrine and epinephrine in neuroendocrine processes. Interestingly, *DBH* mRNA was clearly present in both reproductive and endocrine tissues, specifically in the hypothalamus, pituitary, ovary, and oviduct. It may be, then, that DBH regulates hormone synthesis. DBH modulated the concentration of both progestin receptors and estrogen receptors [11–13], supportive of this suggestion. It is also reasonable that the lungs, which are regulated by the sympathetic nervous system, had high *DBH* expression in our results. This finding also agrees with the results [23, 24]. In human, analysis of *DBH* mRNA had been confirmed high expression in the brain, but also in sympathetically innervated organs, such as lung [23].

The expression of *DBH*, detected with mRNA accumulation, was assessed during the pre-laying, ovulation, oviposition, and broody phases. The expression level drastically fluctuated in reproductive tissues and endocrine tissues. The *DBH* expression levels were high in the ovulation phase and among the lowest observed in the oviposition phase. Similar results were observed in catfish [25]. The fluctuations might be related to the different activity of norepinephrine during the reproductive cycle. Most research focused on DBH regulation of hypothalamo-pituitary-adrenal (HPA) responses [26, 27], but we also found that DBH played a role in hypothalamus-pituitary-gonadal (HPGA) axis. DBH catalyzed a key step in catecholamine biosynthesis, and catecholamine was believed to derive from the extrinsic innervation of the ovary and to participate in the regulation of ovarian development and mature gonadal function [28]. The levels of DBH in hypothalamus or ovary affected luteinizing hormone secretion [10, 29], leading us to speculate that an ovarian steroidogenesis surge, with increased noradrenaline release by the HPGA axis, occurs concomitant with increased *DBH* expression. Interestingly, *DBH* expression was higher in hypothalamus-pituitary than oviduct during the broody phase, while, in contrast, it was higher in the oviduct than the hypothalamus-pituitary during ovulation. We considered this to be evidence for a feedback mechanism that controlled either the enzyme or its gene expression, or both.

We also observed higher DBH expression in Yangzhou geese than in Zhedong geese during the ovulation phase. Yangzhou geese are known for excellent egg-production ability and no broodiness behavior, while Zhedong geese have low egg-production with broodiness behavior. Yangzhou geese might need to release more hormones to ovulate, so the observed higher DBH expression levels might be required to ensure sufficient hormone secretion. The similar results presented in the rat. Stoker TE et al*.* found DBH played an important role in the regulation of the acute effects o on the hormonal control of ovulation [30]. Besides, we found the allele frequency from c.1739 C > T and c.1792 T > C substitutions were significantly different between Zhedong goose and Yangzhou goose. The two substitutions might be associate with egg-production or broodiness behavior.

## Conclusion

In summary, we presented the molecular cloning and characterization of *gDBH* from *Anas cygnoides* and had analyzed its expression during the reproductive cycle. In Zhedong geese, *DBH* expression, as measured by mRNA accumulation, was higher at ovulation than at oviposition. We hypothesized feedback regulation of *gDBH* expression between hypothalamus-pituitary and the oviduct during ovulation and the broody phase. Expression of *DBH* during ovulation was higher in Yangzhou geese than Zhedong geese. Hence this finding provides correlative evidence that *DBH* expression is important in reproduction. Our findings reveal that the gDBH may regulate goose reproductive activity by the HPGA axis.

## Methods

### Animals

All animal experiments were reviewed and approved by the Institutional Animal Care and Use Committee of Yangzhou University. Procedures were performed in accordance with the Regulations for the Administration of Affairs Concerning Experimental Animals (Yangzhou University, China, 2012) and the Standards for the Administration of Experimental Practices (Jiangsu, China, 2008). The two goose breeds used in this study, Zhedong goose and Yangzhou goose, were raised in the breeding farm of Jiangsu Lihua Animal Husbandry Co., Ltd., Changzhou, China, according to the farm’s standard practice. One hundred female geese of each breed were selected randomly for the study. Geese were exposed to natural light and ambient temperature throughout this study and released to an open area during the day, when they were fed ad libitum with rice grain and, when possible, green grass.

### Tissue sample collection

Geese were sacrificed by anesthetizing them with sodium pentobarbital. To investigate *DBH* expression patterns in different tissues, various tissues were removed, immediately frozen in liquid nitrogen, and stored at −80 °C for RNA isolation. These tissues were heart, liver, glandular stomach, lung, spleen, kidney, intestinum tenue, intestinum crissum, cerebrum, cerebellum, muscle, infundibulum of the oviduct, pituitary, hypothalamus, and the stroma of the ovary. A group of Zhedong geese were sacrificed in the pre-laying stage, when they were 120 days old. Three groups of 380-days-old Zhedong geese(5 geese/group) were selected: a laying group with an egg in the oviduct (ovulation, the release of an ovum from a ruptured follicle), a laying group without an egg in the oviduct (oviposition, the laying of the egg), and a brooding group(The goose sits in the nest and the distance between pubic bones is less than two finger widths). Another 5 laying Yangzhou geese with an egg in the oviduct (ovulation) was also selected for comparison with the Zhedong breed.

### Zhedong goose DBH cDNA cloning and sequencing

Total RNA was extracted from collected tissue samples using TRIzol reagent according to the manufacturer’s instruction (TaKaRa, China) and re-suspended in RNase-free water. The concentration and purity were determined with a NanoDrop Spectrophotometer (NanoDrop, USA). After purification, 2 μg of total RNA was reverse transcribed using M-MLV reverse transcriptase (Promega, USA) according to the manufacturer’s protocol. Primers were designed according the unigene (Xu et al., [18]; Additional file 1) and reverse transcription PCR (RT-PCR) was performed using ovarian cDNA from geese. The PCR product was purified, cloned into the pMD19-T vector (TaKaRa, China), and subjected to sequence analysis. The 5′- and 3′-ends of *DBH* were amplified via rapid amplification of cDNA ends (RACE) using the 5´-RACE System for Rapid Amplification of cDNA Ends (Invitrogen, USA) and the 3′-Full RACE Kit (TaKaRa, China), respectively. RACE primers (Additional file 1) were designed using the partial *DBH* nucleotide sequence obtained from RT-PCR. Touchdown and nested PCRs were performed according to the manufacturer’s instructions. Amplicons were then cloned into a plasmid vector for nucleotide sequencing by Sangon Biotech (Shanghai, China).

### Bioinformatics analysis

The Zhedong goose cDNA and deduced DBH amino acid sequences were analyzed using DNAStar (version 7.1). Homology analyses were carried out using Clustal W (http://www.ebi.ac.uk/Tools/msa/). Conserved domains in the protein were identified by the conserved domain database (http://www.ncbi.nlm.nih.gov/Structure/cdd/wrpsb.cgi). A rooted neighbor-joining tree was constructed to determine the phylogenetic relationship using MEGA 6.0 software with 1000 bootstrap replicates to establish the confidence level of each node.

### DBH expression patterns in Zhedong goose and Yangzhou goose

To study expression of the cDNA encoding goose DBH (*gDBH*), we performed real-time quantitative PCR (qPCR) on total RNA isolated from the tissues. Assays were conducted in 20-μL reaction mixes using the SYBR Premix Ex Taq™ (TaKaRa, China) and performed on an ABI two-step RT-PCR system (Applied Biosystems 7500, USA) with diluted first-strand cDNA. The glyceraldehyde-3-phosphate dehydrogenase gene (*GAPDH*) served as an internal reference gene. Quantitative qPCR programs for *DBH* and *GAPDH* were: one cycle of 95 °C for 5 min, 40 cycles of 95 °C for 10 s, 60 °C for 34 s of data collection, and one cycle for the melting curve analysis. All cDNA synthesis reactions were carried out using 100 ng of total RNA per reaction and assayed in three to four technical replicates for each set of biological samples. The same methods were used to determine the *DBH* mRNA expression profile during the reproductive cycle. For the *DBH* mRNA expression profile of the pre-laying Zhedong geese, the chest muscle tissue served as a calibrator. For the differential expression analysis during the reproductive cycle, the oviduct tissue from pre-laying Zhedong geese served as a calibrator. To compare the expression patterns between the Zhedong geese and Yangzhou geese, the mean ΔCt value of the hypothalamus tissue of Zhedong geese within each group was used as the calibrator. Relative expression of mRNA was calculated using the 2^-ΔΔCt^ method [31].

### Identification of SNP on DBH in Zhedong goose and Yangzhou goose

According to the goose genome sequences(scaffold224136, scaffold224137), nine pairs of primers (shown in Additional file 1) were synthesized to identify the polymorphisms. PCR products were amplified from the DNA of Yangzhou geese and Zhedong geese, and sequenced directly by the GenScript Co., Ltd. (Nanjing, China). The obtained sequences were aligned by AlignIR(V2.0) software to screen the potential single nucleotide polymorphisms (SNPs) in the coding region.

### Statistical analyses

Data analysis was performed by using SPSS17.0, then adopted one-way ANOVE analyses to compare the difference among the different tissues, periods and breeds, respectively. Comparisons of genotypes between the different breeds were evaluated by Chi-square (*x*^2^) tests. *P* < 0.01 was considered statistically very significant in all.

## Availability of supporting data

The data sets supporting the results of this article are included within the article and its additional files. The cDNA sequence of DBH from Zhedong goose been deposited in the GenBank of National Center for Biotechnology Information (NCBI) with accession number KU672379.

## Additional file

Additional file 1:
**Primers used in this study.** (DOCX 20 kb)

## Abbreviations

DBH: dopamine β-hydroxylase
RACE: rapid amplification of cDNA ends
RT-PCR: reverse transcription PCR
UTR: untranslated region
