# Supplementary material for: The dopamine β-hydroxylase gene in Chinese goose (Anas cygnoides): cloning, characterization, and expression during the reproductive cycle
Source: BMC Genet. 2016 Feb 24;17:48. doi: 10.1186/s12863-016-0355-8 (PMC4766643; doi:10.1186/s12863-016-0355-8)
Supplement: Additional file 1: — Primers used in this study. (DOCX 20 kb) [file 12863_2016_355_MOESM1_ESM.docx]

**Additional file 1: Primers used in this study**

| Primer name | Oligo sequences (5'–3') | Type |
| --- | --- | --- |
| cDBHF  cDBHR | CCCGTGGCACCTATTTTCCCTAT  GTTTTAGCCCTGGCCTGGAGTTT | ORF amplification |
| GSP1  GSP2  GSP3 | CTGGGGATAACGACAC  GTCTTCATGTCGCTGGGCAG  GGGATGGTGATGTTGGGCTT | 5′RACE |
| 3′Router  3′Rinner | TACTTCAGCCTCGTGAACAGGTT  GGAACACCTTCAACAGAGACGTG | 3′RACE |
| eDBH-F  eDBH-R | ACGCCAAGATGAAGCCAGA  AGTGAATCTCAAGGCGCAGA | Expression proﬁle |
| GAPDH-F  GAPDH-R | GGTGGTGCTAAGCGTGTCAT  CCCTCCACAATGCCAAAGTT | Expression proﬁle |
| Exon1-F  Exon1-R | TAAGAGCAAATAACATCAGCCC  TGGACCACTTTCATTGAGCAG | Polymorphism |
| Exon2-F  Exon2-R | TACACCCAGAACAACGCAGAC  AGCCATCCCAAAAAACACAAC | Polymorphism |
| Exon3-F  Exon3-R | ATTCACACTCTCTGGTATCCT  TTCTCCTCTTTCACCTCTTCT | Polymorphism |
| Exon4/5-F  Exon4/5-R | CTTGGCAAACGGTCCTAACAT  GCAGAGTGGCAGTCCCTATCA | Polymorphism |
| Exon6-F  Exon6-R | TCATTTTTCACACCTGACCTTACC  AGCCAATCTTTGCGTTTCCATA | Polymorphism |
| Exon7-F  Exon7-R | CGAAAGAAGTCGTCGGTGC  TCCTGAGAGGGGGGAGAAA | Polymorphism |
| Exon8/9-F  Exon8/9-R | TGCCTTTTTCTCCCCCCTCTCA  TCGCTTCCTTGCCACCCTTCTC | Polymorphism |
| Exon10-F  Exon10-R | GAGTGTGCGTGACGGTGTGTGT  CTTTTTTTCCAGCATTGCCAGC | Polymorphism |
| Exon11-F  Exon11-R | CTTTCTGGGTATGGGACTTC  GACAGCCCTGCTTTAACACT | Polymorphism |
